# Supplementary material for: Mechanisms governing the pioneering and redistribution capabilities of the non-classical pioneer PU.1
Source: Nat Commun. 2020 Jan 21;11:402. doi: 10.1038/s41467-019-13960-2 (PMC6972792; doi:10.1038/s41467-019-13960-2)
Supplement: Supplementary file 7 — Source data [file 41467_2019_13960_MOESM7_ESM.zip › Source_Data/Figure6/Figure6B_MotifScanOutput/FLI1_mutPU.1/homerResults/motif13.similar.html]

motif13

## Information for motif13

G
A
T
C
T
G
C
A
C
A
T
G
T
G
A
C
A
G
C
T
T
C
A
G
A
G
C
T
G
C
A
T
C
G
A
T
A
T
G
C
G
T
A
C
  
Reverse Opposite:  

C
A
T
G
A
T
C
G
G
C
T
A
C
G
T
A
T
C
G
A
A
G
T
C
T
C
G
A
A
C
T
G
G
T
A
C
A
C
G
T
C
T
A
G
  

|  |  |
| --- | --- |
| p-value: | 1e-90 |
| log p-value: | -2.081e+02 |
| Information Content per bp: | 1.666 |
| Number of Target Sequences with motif | 909.0 |
| Percentage of Target Sequences with motif | 8.86% |
| Number of Background Sequences with motif | 1409.4 |
| Percentage of Background Sequences with motif | 3.70% |
| Average Position of motif in Targets | 102.1 +/- 52.7bp |
| Average Position of motif in Background | 97.4 +/- 68.2bp |
| Strand Bias (log2 ratio + to - strand density) | -0.1 |
| Multiplicity (# of sites on avg that occur together) | 1.06 |
| Motif File: | file (matrix) reverse opposite |

### Similar de novo motifs found

|  |  |  |  |  |  |  |  |
| --- | --- | --- | --- | --- | --- | --- | --- |
| Rank | Match Score | Redundant Motif | P-value | log P-value | % of Targets | % of Background | Motif file |
| 1 | 0.924 | A G T C C G T A A C T G T G A C G A C T A T C G A G C T C G A T C A G T A T G C A G T C C A G T | 1e-68 | -156.795858 | 1.34% | 0.06% | motif file (matrix) |
| 2 | 0.954 | T G C A T G A C C T A G T A C G T G C A T C G A T C G A A G T C T C G A A C T G T G A C C G A T C A T G | 1e-54 | -124.689782 | 1.81% | 0.29% | motif file (matrix) |
| 3 | 0.801 | C G T A A G T C G T C A A C T G A G T C A C G T A C T G | 1e-43 | -100.173202 | 20.57% | 14.75% | motif file (matrix) |
